# Supplementary material for: Climate change exacerbates hurricane flood hazards along US Atlantic and Gulf Coasts in spatially varying patterns
Source: Nat Commun. 2019 Aug 22;10:3785. doi: 10.1038/s41467-019-11755-z (PMC6706450; doi:10.1038/s41467-019-11755-z)
Supplement: Supplementary file 1 — Supplementary Information [file 41467_2019_11755_MOESM1_ESM.pdf]

Climate Change Exacerbates Hurricane Flood Hazards Along U.S. Atlantic and Gulf Coasts in Spatially-varying Patterns

Marsooli et al.

Supplementary

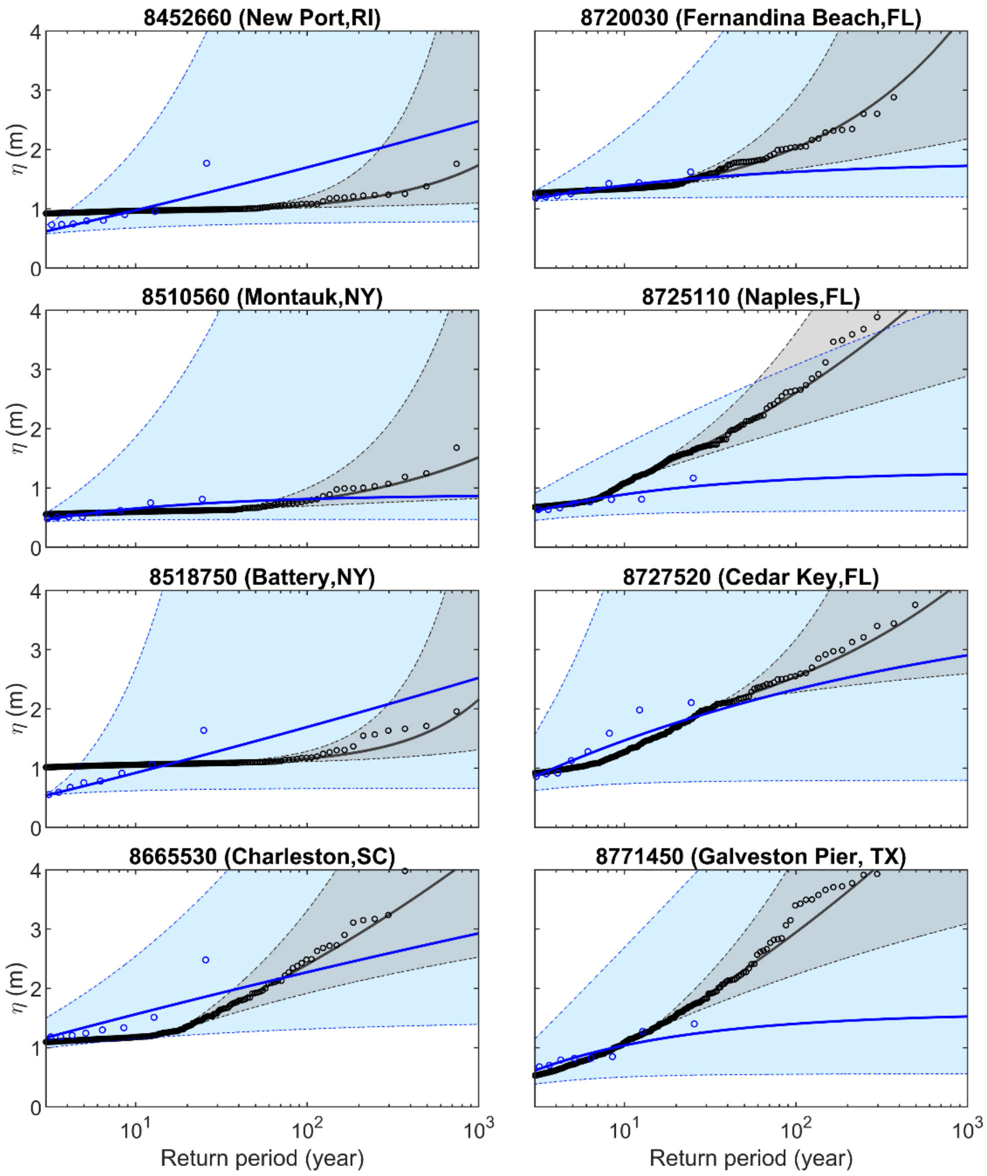

Supplementary Figure 1. Flood return period curves estimated based on the observed (blue) and modeled NCEP–based (black) storm tides for the historical period of 1980–2005. Each circle represents the peak storm tide associated with a TC event. The shading shows the 90% confidence interval. Source data are provided as a Source Data file.

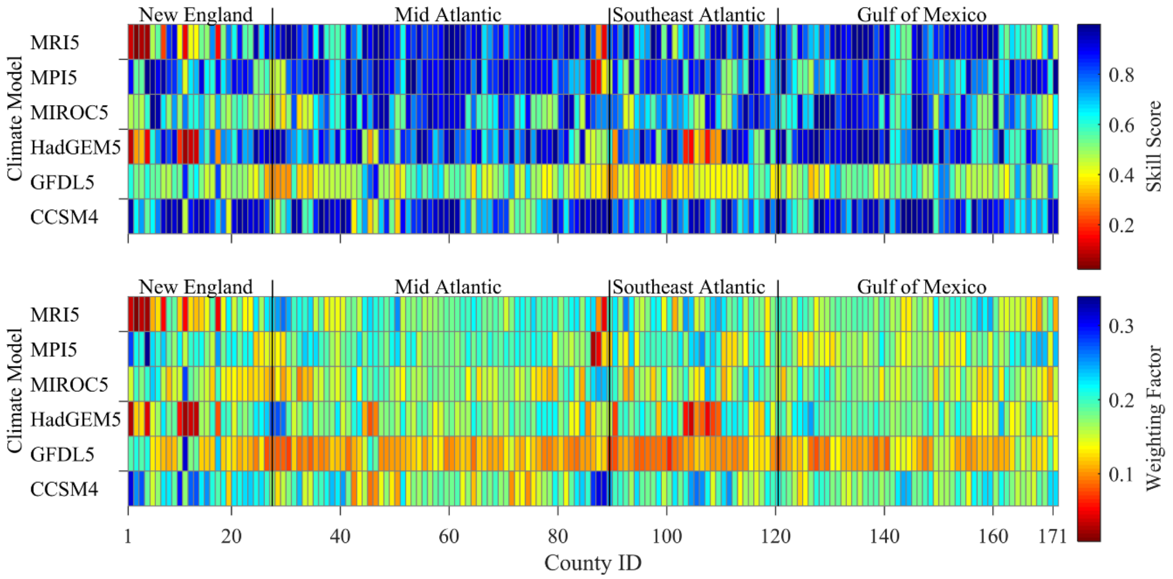

Supplementary Figure 2. Skill score (top) and weighting factor (bottom) of each climate model calculated by comparing the NCEP–based and climate–model–based storm tide return levels for the historical period of 1980–2005. Weighting factors are separately calculated for each coastal county. Source data are provided as a Source Data file.

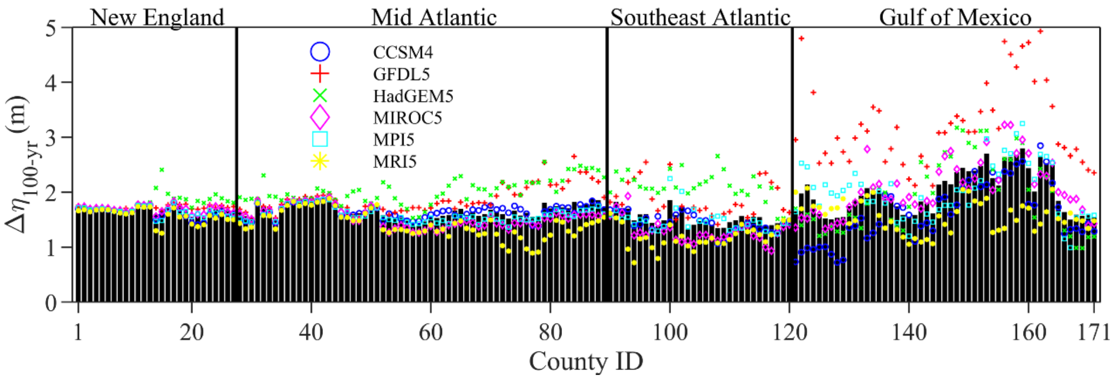

Supplementary Figure 3. Projected weighted–average (black bars) changes in the best estimate of $\eta_{100\text{-yr}}$ from the historical period to the future period. Projections from induvial models are also shown. Source data are provided as a Source Data file.

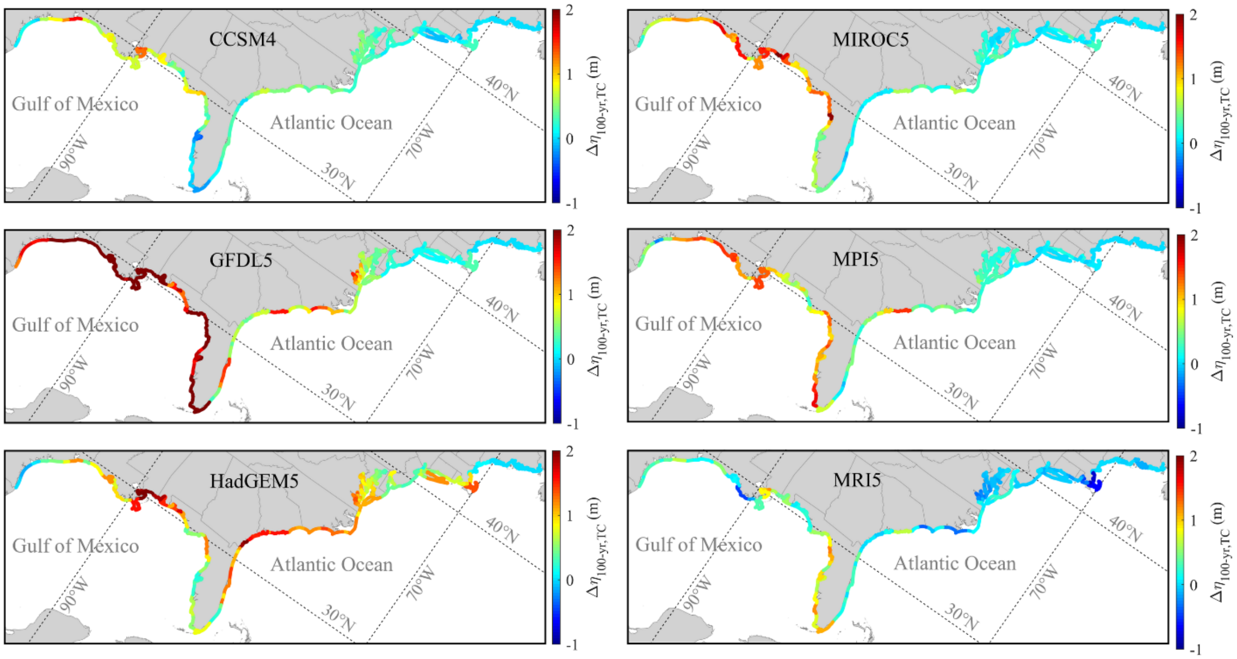

Supplementary Figure 4. Changes in the best estimate of $\eta_{100\text{-yr}}$ induced by only TC climatology change (i.e. in the absence of SLR) for the future period of 2070–2095. Source data are provided as a Source Data file.

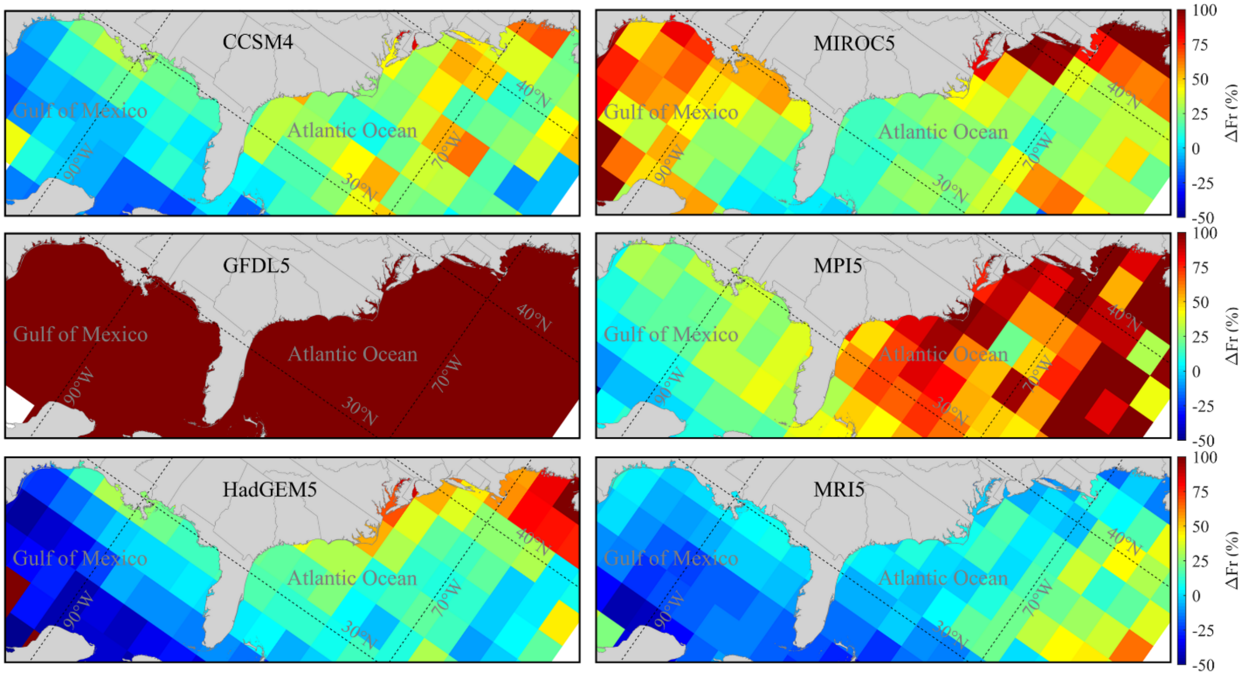

Supplementary Figure 5. Changes in TC annual frequency Fr projected by six climate models for the future period. Results in each box (2° by 2° resolution) represent 90th percentile of storms that pass through the box. Source data are provided as a Source Data file.

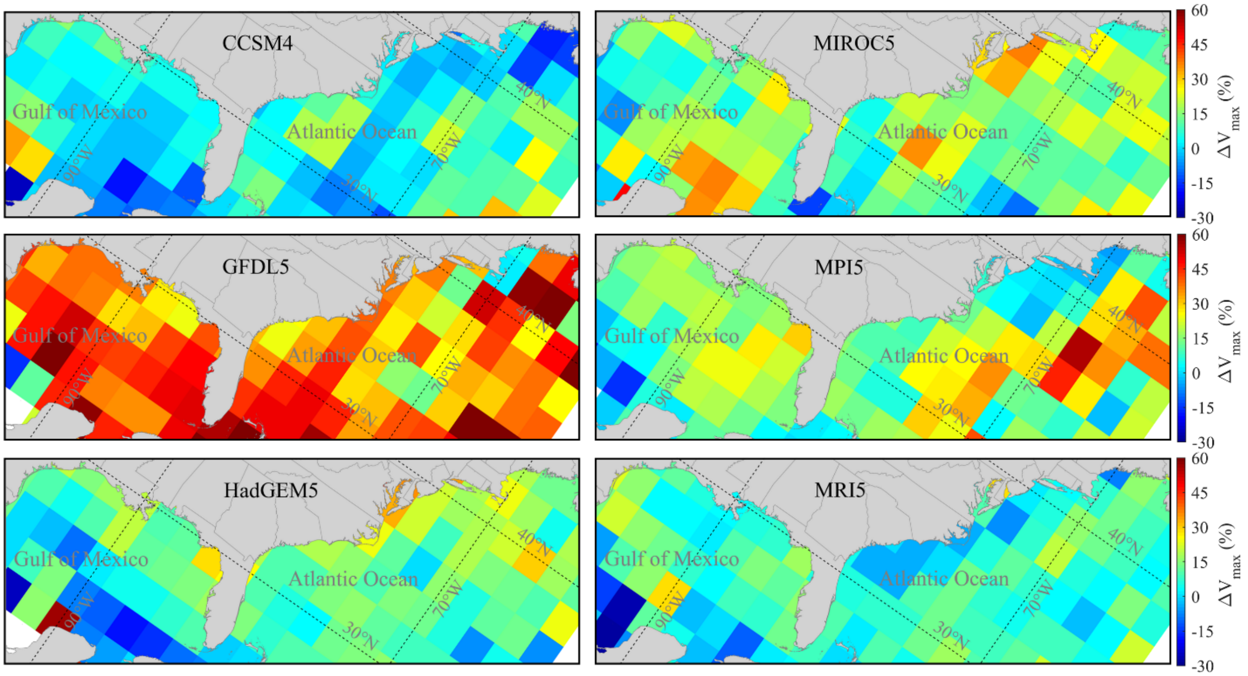

Supplementary Figure 6. Changes in maximum wind speed V_{max} projected by six climate models for the future period. Results in each box (2° by 2° resolution) represent 90th percentile of storms that pass through the box. Source data are provided as a Source Data file.

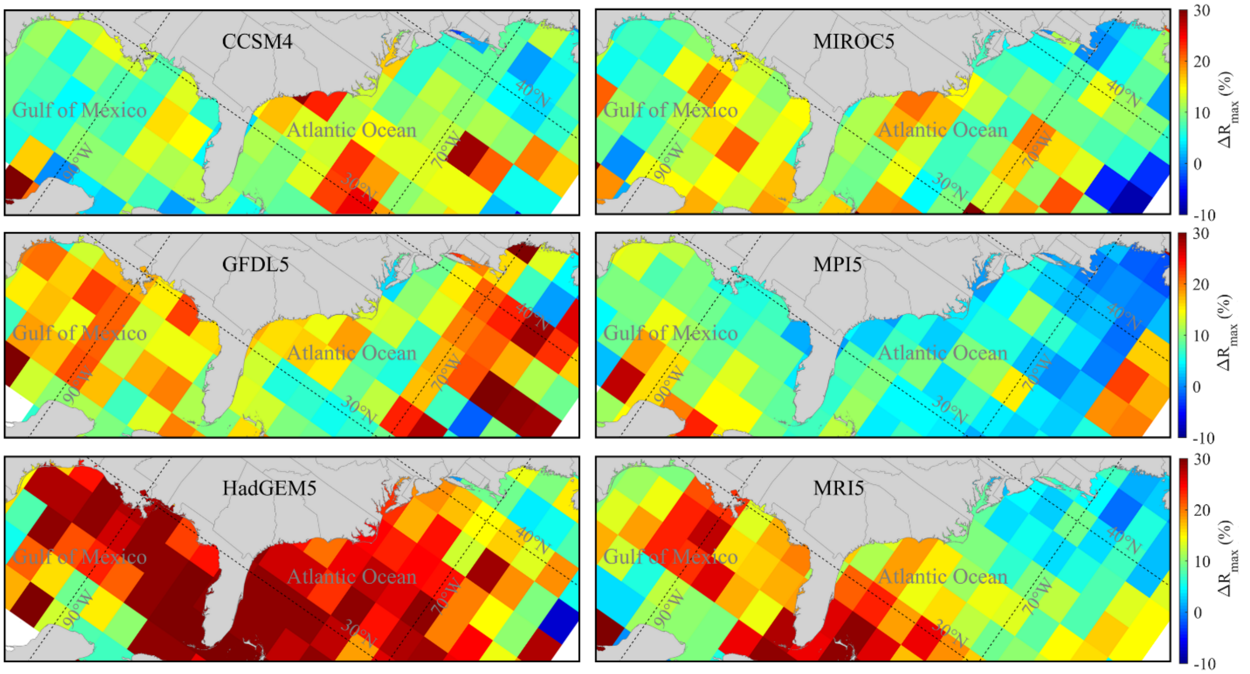

Supplementary Figure 7. Changes in radius of maximum wind speed R_{max} projected by six climate models for the future period. Results in each box (2° by 2° resolution) represent 90th percentile of storms that pass through the box. Source data are provided as a Source Data file.

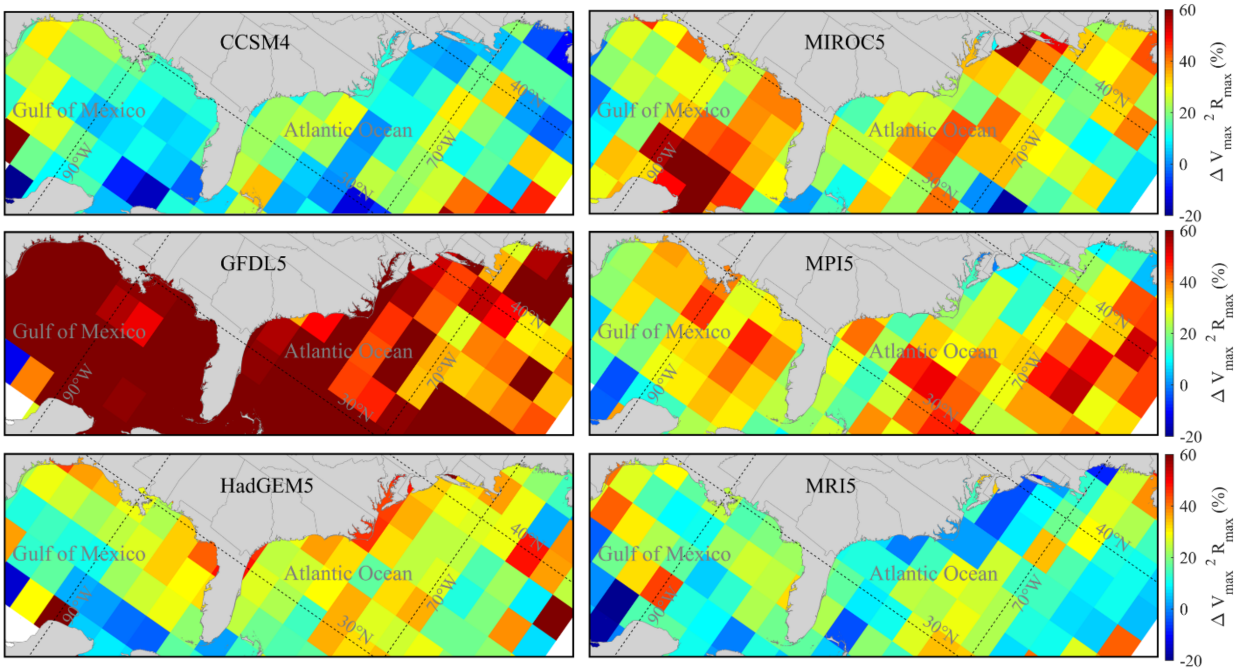

Supplementary Figure 8. Changes in the index $V_{max}^2R_{max}$ projected by six climate models for the future period. Results in each box (2° by 2° resolution) represent 90th percentile of storms that pass through the box. Source data are provided as a Source Data file.
